# Integrating private health facilities in government-led health systems: a case study of the public–private mix approach in Ethiopia

**DOI:** 10.1186/s12913-022-08769-7

**Published:** 2022-12-03

**Authors:** Disha Ali, Asfawesen Gebre-Yohannes Woldegiorgis, Mesfin Tilaye, Yonas Yilma, Hanna Y. Berhane, Dagmawit Tewahido, Gebeyehu Abelti, Rachel Neill, Ndeye Silla, Lauren Gilliss, Mahua Mandal

**Affiliations:** 1 John Snow, Inc. (JSI), Arlington, VA USA; 2 Private Health Sector Project, Abt Associates, Addis Ababa, Ethiopia; 3 USAID/Ethiopia, Entoto Street, Addis Ababa, Ethiopia; 4 Independent Consultant, Addis Ababa, Ethiopia; 5 Addis Continental Institute of Public Health, Ayat, Addis Ababa, Ethiopia; 6 Independent Consultant, Rockville, MD USA; 7 Independent Consultant, Baltimore, MD USA

**Keywords:** Ethiopia, Public–private mix, Health systems, Private health sector engagement, Health systems building blocks, Policy, Private health facilities

## Abstract

**Background:**

Private health care facilities working in partnership with the public health sector is one option to create sustainable health systems and ensure health and well-being for all in low-income countries. As the second-most populous country in Africa with a rapidly growing economy, demand for health services in Ethiopia is increasing and one-quarter of its health facilities are privately owned. The Private Health Sector Program (PHSP), funded by the United States Agency for International Development, implemented a series of public–private partnership in health projects from 2004 to 2020 to address several public health priorities, including tuberculosis, malaria, HIV/AIDS, and family planning. We assessed PHSP’s performance in leadership and governance, access to medicines, health management information systems, human resources, service provision, and finance.

**Methods:**

The World Health Organization’s health systems strengthening framework, which is organized around six health system building blocks, guided the assessment. We conducted 50 key informant interviews and a health facility assessment at 106 private health facilities supported by the PHSP to evaluate its performance.

**Results:**

All six building blocks were addressed by the program and key informants shared that several policy and strategic changes were conducive to supporting the functioning of private health facilities. The provision of free medicines from the public pharmaceutical logistics system, relaxation of strict regulatory policies that restricted service provision through the private sector, training of private providers, and public–private mix guidelines developed for tuberculosis, malaria, and reproductive, maternal, newborn, child, and adolescent health helped increase the use of services at health facilities.

**Conclusions:**

Some challenges and threats to sustainability remain, including fragile partnerships between public and private bodies, resource constraints, mistrust between the public and private sectors, limited incentives for the private sector, and oversight of the quality of services. To continue with gains in the policy environment, service accessibility, and other aspects of the health system, the government and international communities must work collaboratively to address public–private partnerships in health areas that can be strengthened. Future efforts should emphasize a mechanism to ensure that the private sector is capable, incentivized, and supervised to deliver continuous, high-quality and equitable services.

## Background

To improve universal health coverage (UHC) and attain the Sustainable Development Goals, countries must work with different partners toward a common goal of improving population health [1, 2]. The role of the private sector in healthcare provision in low- and middle-income countries (LMICs) is widely recognized and increasingly considered a component for achieving UHC [3, 4]. Recent analysis indicates that “the private sector provides nearly 40% of all healthcare in the Pan American Health Organization (PAHO), African Regional Office (AFRO), and Western Pacific Regional Office (WPRO) regions, 57% in South-East Asia Region (SEARO), and 62% in Eastern Mediterranean Region (EMRO)” [5]. In Africa, 52% of outpatient care seeking occurs in the private sector, including private, for-profit providers, shops, and the informal sector [5]. Literature from HIV/AIDS, routine childhood immunization, child health, malaria, and tuberculosis (TB) highlights the potential of leveraging existing private health facilities (PHFs) to expand access to essential services [6–11].

Despite acknowledgement of the importance of PHFs in care provision, specific policies and mechanisms to effectively engage them are lacking [12]. The diversity of the private health sector creates challenges to developing a global evidence base [13]. Many existing approaches, including contracting, social franchising, voucher schemes, accreditation, and commodity social marketing, are focused on individual providers [14]. There is less knowledge about interventions at the macro-level to engage PHFs [15], and limited understanding of how governments can provide effective stewardship [16, 17].

### Study setting

Ethiopia has achieved impressive milestones in improving the health of its population—increasing child survival, reducing maternal mortality, and improving overall life expectancies [18–20]. Despite these gains, the country has pressing health issues: its maternal mortality ratio is still high at 412 per 100,000 live births [21]; and infectious diseases, such as HIV/AIDS, TB, and malaria, contribute to 85% of Ethiopia’s disease burden [18, 22]. Although the public sector remains the dominant care provider, demand for private healthcare services is also increasing [23]. Twenty-seven percent of health facilities are privately owned, with approximately 25% of outpatient visits and 20% of inpatient visits taking place at a PHF [23].

Public–private partnerships in health (PPPH) are agreements that allow governments and private sector actors to collaborate to address health issues [24, 25]. These partnerships have the potential to improve health service quality and capacity, regulation, and innovation [25]. The Government of Ethiopia (GOE) has demonstrated political commitment to improving the engagement of the private sector in the provision of health services. With support from partners, the Ministry of Health (MOH) established a PPPH Unit in its Partnership and Collaboration Directorate in 2018 to strengthen the implementation of the 2013 PPPH strategic framework [26]. The GOE Health Sector Transformation Plan II for 2021–2025 stated that "Strengthening the engagement of the private sector in the health sector priorities is a major strategic area" [27].

In support of these objectives, the United States Agency for International Development (USAID) supported Abt Associates and its partners in implementing the Private Health Sector Program (PHSP) from 2004 to 2020. PHSP’s strategy focused on developing and implementing the public–private mix (PPM) model to contribute to the mitigation of the impact of diseases of public health importance. PPM partnerships are generally implemented for short periods, are non-contractual, and focus on strategic collaboration for vertically addressing a disease [28]. Evidence from PPM partnerships comes largely from TB programming, which suggests that it has potential to increase diagnosis and improve value for money [29] and has been expanded to address malaria diagnosis and treatment [30–32]. Starting in 2004 with a PPM Directly Observed Treatment Short Course (DOTS) strategy for the TB control program and HIV/AIDS [33, 34], the program expanded to more private facilities and incorporated malaria, HIV/AIDS, maternal, newborn, and child health (MNCH), and family planning (FP) [34].

The PHSP facilitated and supported the development of PPM guidelines in these priority health areas to serve the purpose of building a strong PPPH. The PHSP’s role was to work as a an external broker, building collaboration among different government institutions, including the MOH, regional health bureaus (RHBs), and PHFs; defining responsibilities among the partners; and formalizing the partnership through memorandums of understanding (MOUs) [34]. Support was provided in eight regions (Afar, Amhara, Beninshangul-Gumuz, Gambella, Oromia, Southern Nations, Nationalities and Peoples Region, Tigray, and Addis Ababa) from 2015 to 2020. In 2020, USAID commissioned Data for Impact (D4I) to conduct an endline evaluation of the PHSP program’s third phase, from 2015–2020, to inform future support for public–private health sector initiatives in Ethiopia [35].

### Analyzing a PPPH initiative through the health systems building blocks

Assessment and evaluation of PPPH have identified areas of challenges for public–private collaboration, including inadequate policy framework and ineffective stakeholder engagement, as well as potential benefits such as enhanced efficiency and effectiveness of health service delivery [24, 25, 36, 37]. A strong health system creates a sustainable and robust foundation for PPPH [15, 38, 39]. Health system characteristics impact the relative performance of PHFs, including how the private sector fills existing gaps in the public sector and the effectiveness of regulation that shapes a PHF’s functioning [13, 15]. However, there is lack of evidence about how system factors shape PHF performance or how policy instruments can be used to influence the PPM toward UHC objectives [13, 15].

The World Health Organization’s (WHO) health systems strengthening framework identifies six health systems building blocks: leadership/governance, financing, service delivery, health workforce, health information systems (HMIS), and access to essential medicines [40]. This framework has been applied to understand the preparedness, performance, and gaps in the health systems in African countries in the context of the Sustainable Development Goals. It is essential to create a sustainable, robust foundation for the PPPH to grow and flourish while grounded within a strong health system [38, 39]. Elements required for successful PPPHs are similar to the elements needed for strong health systems. As such, it is useful to assess the PPPH through the lens of the health system building blocks, and this framework was used to guide the assessment of a PPPH implemented in Ethiopia.

### Study objective and contributions

This study retrospectively assessed the performance of the Private Health Sector Program using the six WHO health systems building blocks as an analytical framework. The PHSP offers a case study for exploring the challenges and opportunities of integrating PHFs in government-led health systems to improve service coverage of essential health services in a low-income country health system context. Findings from this study aim to generate implementation insights for strengthening PPPHs and the PPM in LMICs.

## Methods

The evaluation design was a retrospective, mixed-method, cross-sectional assessment conducted at program endline without a comparison group. Data sources included qualitative interviews with key informants and a health facility assessment of selected PHFs.

### Sample size, sampling, and characteristics of participants

#### Key informant interviews

We purposively selected stakeholders at national, regional, and facility levels as key informants who were familiar with the PHSP’s activities and were involved in the program and PPM processes. National-level interviews included MOH representatives from directorates involved in priority areas of PHSP support (TB, malaria, MNCH and FP); government and regulatory bodies, such as the Ethiopian Food and Drug Administration (EFDA) and Ethiopian Pharmaceutical Supply Agency (EPSA); and professional associations, such as the Ethiopian Medical Laboratory Association (EMLA). Additional key informants were advisors from the PHSP who provided technical support and guidance to the MOH and RHBs in the implementation of PPMs. At the regional level, we interviewed representatives from RHBs in Tigray, Amhara, Oromia, Addis Ababa, Afar, and Harari; PHF owners; and representatives of regional PHF associations (PHFA). Health facility owners covering different service sectors (TB, malaria, MNCH and FP) were interviewed to understand their perspectives and experiences implementing the PPM. A total of 50 key informant interviews (KIIs) were conducted (Table 1).

**Table 1:** KIIs conducted, by category of informant

Type of Key Informant	Number of Interviews Conducted
MOH	5
RHB	6
PHFA	6
PHSP staff	6
PHFs	18
Development Credit Authority (DCA)	2
DCA beneficiaries	3
Federal regulatory bodies	4
Total KIIs	50

#### Endline health facility assessment of PHSP-supported PHFs 

The health facility assessment (HFA) collected data from 106 of the 332 PHFs supported by the PHSP in 2020. The PHFs were purposefully selected to represent all PHSP regions, types of health facilities, and service delivery technical areas (TB, malaria, and FP) (see Tables 2 and 3). The sample of facilities covered seven regions and Addis Ababa and was proportional to the total number of PHFs in each service area. PHSP supported a higher number of PHFs from Oromia, Addis Ababa, and Amhara, and these regions yielded a higher percentage of PHFs in the selected sample. Fewer than 10 PHFs from Benishangul-Gumuz, Afar and Gambella received support from PHSP, and we selected 4–5 PHFs from these regions to ensure representation.

**Table 2:** Sampled health facilities, by region (*n* = 106)

Region	Number (percent) of health facilities
Addis Ababa	21 (19.81)
Afar	5 (4.72)
Amhara	21 (19.81)
Benishangul-Gumuz	4 (3.77)
Gambella	4 (3.77)
Oromia	27 (25.47)
Southern Nations, Nationalities and People Region	13 (12.26)
Tigray	11 (1.38)
Total	106 (100.00)

**Table 3:** Sampled health facilities, by service delivery technical area (*n* = 106)

Technical Area	Number (percent) of health facilities
TB	75 (70.75)
Malaria	50 (47.17)
FP	54 (50.94)
MNCH	36 (33.96)

^*^Note: Some PHFs provided services in more than one technical area

Some health facilities received PHSP support in more than one technical area and therefore provided more than one type of technical service. For those health facilities, all of the services of interest in this study are included in the sample. Table 2 presents the percentage distribution of selected PHFs by region, and Addis Ababa.

### Data collection, management, and analysis

D4I collected data in partnership with the Addis Continental Institute of Public Health (ACIPH) between July and October 2020. Most data were collected over the phone and digitally recorded.

#### KIIs

The data collection team administered an open-ended, semi-structured interview guide appropriate for different types of stakeholders. The interview guide included questions on the improvements from and contribution of the program, challenges, and gaps; issues of sustainability in the policy environment; service delivery and service utilization; and the overall functioning of the private healthcare system. A team of six interviewers experienced in qualitative research received orientation on the data collection tools, conducted phone interviews in Amharic, took notes, and recorded the interviews with participant consent and permission. The interviewers also conducted some in-person interviews in English, mainly with PHSP staff and government bodies, with proper COVID-19 precautions. If any interviews needed further clarifications, the interviewees were reached through follow-up calls. The qualitative research team translated and transcribed all interviews in English.

Transcripts were analyzed using a coding framework based on the program’s purpose, content of the interview guides and the application of broad key themes (achievements, challenges, and gaps). We coded and analyzed the texts by type of respondents and thematic areas to group and identify patterns. The research team discussed iterations of the framework during the coding process as well as evolving themes and data saturation and any interpretations of translations that seemed vague.

#### HFA

A pretested, structured questionnaire programmed onto a tablet was used to collect quantitative data for the HFA using Open Data Kit software. A team of six research assistants with medical backgrounds conducted phone interviews and recorded the responses on the tablets. In most cases, the respondents needed to review their records for information on stock-outs and number of trained personnel, and the data collectors called them a second time to retrieve this information. Additionally, the study team requested that HFA respondents take pictures of facility records and send them via telegram, and about half the health facilities did so.

The PHFs in the sample responded to questions about the services they provided. We selected key indicators aligned with the thematic areas of support provided to the health systems, including training, supervision, and the availability of medicines. Descriptive analyses were conducted in Stata to assess the status of the health systems-related indicators.

#### Trustworthiness

This study was carried out in mid-2020, at the height of the first wave of COVID-19 restrictions. Data collection had to be carried out remotely due to the travel constraints and mandatory lockdowns enforced during the pandemic. As a result, KIIs were scheduled and conducted as telephone interviews. A minor proportion (< 10) of interviews were carried out in-person. Likewise the HFA questionnaires were carried out by telephone. Clarifications were confirmed through follow-up calls to participants, as needed. While a typical HFA often includes the observation of services and patient flow and the visual inspection of drug and equipment inventories, this was not possible as a remote study. Given these limitations, we adapted the HFA approach to seek information on key performance indicators and to verify reported data remotely.

We also used triangulation across different methods and participants to further strengthen the credibility of the study’s findings. Triangulation allowed the emergence of comparisons and patterns that were important to our analysis. The main instruments consisted of closed-ended and open-ended HFA questionnaires and semi-structured, in-depth interview guides. Respondents included a range of stakeholders from national, regional and health facility levels and included perspectives from policymakers, facility owners, managers and health workers. These different ways of gathering information from different types of respondents allowed a comparison and verification of responses, which increased the validity and dependability of the data.

Finally, the evaluation that formed the basis of this study was carried out by an independent, external organization that followed best practices in sampling and remote data collection processes, including providing a clear rationale for the sampling design and sampling frame, determination of qualitative data saturation, and maintaining ethics in the research design. An independent evaluation increases the likelihood of an unbiased performance assessment and enhances the credibility of the findings.

## Results

Results from the KIIs and HFA are presented jointly, organized by health systems building block. PHSP’s support toward HIV stopped in 2018 due to the termination of funding by the donor. As such, the assessment did not focus on HIV. In addition, PHSP was involved in MNCH for less than two years due to the late initiation and early ending of the program [34]. Due to the short engagement on MNCH, this paper includes some findings from the stakeholders’ interviews at the policy level on MNCH but excludes results from the HFA.

### Leadership and governance

#### Development of policies to support PHFs in the overall health system

The MOH and regional health system levels demonstrated leadership through the involvement of RHBs and woreda health offices in support of PHFs for the provision of TB, malaria, HIV/AIDS, and MNCH services. The MOH strengthened the private health system by formulating policies and strategies toward a PPPH, including initiating a form of PPP, PPM-DOTS, in 2004. In 2018, the MOH established a full unit of PPPH within the Partnership and Collaboration Directorate to strengthen the implementation of the 2013 PPPH strategic framework. The MOH developed and revised the PPM for TB twice and developed a PPM for malaria and for MNCH and FP between 2004 and 2020, with extensive technical support from partners, especially from the PHSP.

The commitment and involvement of RHBs were tangible through their direct support to the PHFs. For example, RHBs made an autonomous decision to provide logistics and technical support to PHFs for HIV/AIDS services, even though the MOH did not have any PPM implementation guidelines and the donor and PHSP’s support ceased for HIV/AIDS. Key informants from the PHSP and RHBs perceived that the MOH’s commitment and leadership was the culmination of several converging factors: increasing demand for health care services from the private for-profit and non-profit sectors [41]; persistent and ongoing advocacy; technical assistance from partners, including the PHSP and Clinton Health Access Initiative (CHAI); and evidence of the initial success of PPM-DOTs, all contributed to policies that were inclusive of PHFs. A MOH senior respondent summarized:

The public-private partnership is one of the pillars in [the] Health Sector Transformation Plan…to create an enabling environment for the private sector to invest in health. The private health sector is cross-sectoral and goes beyond the health sector; there is a PPP [public-private partnership] proclamation, policy and direction, and implementation guideline[s]...approved by the Ministry of Finance. These are ventures where the private and public sectors can mobilize their resources and invest for improving the health system.

##### Remaining gaps

The federal and regional governments were not yet able to formulate private sector engagement endeavors to implement PPPH and PPM policies effectively and independently on a large scale without technical and financial support from donors. Some stakeholders perceived a disconnect between the PPPH Unit and the overall MOH because the Unit had not been incorporated in the MOH organogram to support private sector engagement for health, and no specific government resources were allocated. Another disconnect was the vertical establishment of PPMs. With support and advocacy from partners, such as the PHSP, different directorates developed PPM guidelines for individual diseases. For example, as of 2020, PPM guidelines had been finalized for TB, malaria, and MNCH and FP; however, these MOH directorates did not have a formal linkage to the PPPH Unit, exposing a weakness in the system.

Another gap was the absence of a dedicated person or unit for PPM at the RHB level. Although the PPM programs and private sector engagement were operationalized at the regional level and below, the lack of a formal position was a major gap. It was also revealed that the private sector was not included in regional planning or budget allocation processes. One PPPH Unit representative described:

The PPMs currently focused on free social franchising programs; there is a lack of understanding of the concept of other modalities of PPPH among the authorities…Although we have more than a decade of experience in PPPH, our experience is limited to free public health services.

#### Revisions to regulatory standards

The regulatory standards previously in place were considered too stringent and not conducive for service delivery by PHFs. One high-level MOH respondent who was familiar with the standards described*:*

The previous requirements recommended by the FMHACA [Food, Medicine and Health Care Administration and Control Authority] were very rigid and not suitable for the Ethiopian context (for example, minimum number of staff per service) and it didn’t have the incentives to encourage private facilities.

The PHSP and representatives from PHFA coordinated with the MOH and the FMHACA (later named the EFDA), the lead regulatory agency for commodity quality standards. This advocacy resulted in the government revising several service quality standards to ensure that the regulatory standards were realistic, transparent, feasible, and applicable to both public and PHFs [35].

Relaxing the regulatory standards did not imply a relaxation of monitoring of compliance with the standards by PHFs and periodic checking of licensing standards. With support from the PHSP, the FMHACA/EFDA developed and implemented a modernized health standards monitoring system with digital equipment connected to a server. Based on the inspections, the RHBs exercised full authority for regulatory oversight and for licensing and relicensing of all health facilities at the regional level. An RHB representative stated:

We conduct supervisions as per a standardized checklist. For example, we come across health facilities that don’t adhere to the TB infection prevention guidelines. [….] We give feedback and discuss with the owner if the standards are not met. Based on the supervision outcomes, there were facilities that might be disallowed to provide specific services. We try to strengthen these facilities.

##### Remaining gaps

Despite improvements in the partnership between the RHBs and PHFs, some stakeholders indicated that there were still ongoing tensions around the implementation of regulatory standards. One regulatory representative expressed concern that bias against PHFs might lead to double standards in actions for the inspection of private and public health facilities. Regulators could cancel licenses or take other administrative actions against PHFs if they deviated from the standards, but they could not take any action against public health facilities for the same deviation. Some PHF respondents and regulators alluded to this issue of double standards, with one regional regulatory body expressing that:

It is easy to regulate the private health sector and take action against them for not following the standard, but when you come to the government facilities you can’t control them. Sometimes the authority doesn’t accept the issues with the public health centers, as it may lead to conflict, so the control is focused on the private sector.

#### Trust between public and private health sectors

Historically, there has been deep-rooted mistrust at both MOH and RHB levels toward the private sector. Key informants, especially those from the PHSP team, indicated that increased trust and better working relationships between the government and PHFs had evolved slowly over time, partly a result of the PHSP’s persistent advocacy between the MOH and relevant stakeholders to create a more positive and trustworthy partnership. Regional-level stakeholders expressed the view that engaging RHBs and local health offices in advocacy, the signing of MOUs, and the implementation of PPM had helped shift some RHB staff’s perceptions about working with the private sector.

Making regulatory standards more transparent and less punitive for the private sector, delivery of services, and continuous involvement and support through regular interactions and supervision by the RHBs also contributed to establishing a baseline level of trust, especially between the RHBs and PHFs. One PHF owner explained the relationship as follows:

Government used to consider the private sector as just organizations established only for profit, hence there was no interaction between those bodies. People come for our services and they make the decision for their own benefit. The negative view has been changing.

##### Remaining gaps

Despite improvements, mistrust between the public and private health sectors persists. Some key informants mentioned that there was reluctance among certain professional groups at the MOH, including a pharmacy association, to shift tasks, such as dispensing antiretrovirals or anti-malaria medications from pharmacies to nurses. Some RHB representatives shared their belief that because PHFs were profit-oriented, they would not look after the best interests of their clients.

One of the major concerns expressed by the public sector stakeholders was that PHFs might misuse the subsidized or free medicines by seeking financial benefits instead of providing the medications to clients for free or at a minimal cost. One RHB representative expressed:

There is a low commitment of facility owners to keep providing the service since these services are not profitable. Both TB and malaria medications and laboratory services are free according to the country’s law. But sometimes, patients are asked to buy the medicine from outside. They [PHF] also charge high for laboratory services.

### Access to essential medicines

#### Incorporation of PHFs in the government’s integrated pharmaceutical logistics system

The integrated pharmaceutical logistics system (IPLS) strategy ensured that the PHFs that signed MOUs with the RHBs would get government-purchased, free medicines, supplies, and reagents for TB, malaria, and FP services through the EPSA’s distribution system. The national IPLS also included the PHFs in training manuals and the accurate estimate of medicine requirements by the PHFs in the national supply chain and stock level quantification process in 2017 [21].

The importance of having the provision of free or affordable medicines is part of the PPM guidelines. For example, a stakeholder from the national malaria unit of the MOH said*:*

We have seen some hope for malaria elimination which is our goal. The PPM guideline provides guidance and approval to the PHFs to give malaria treatment free of charge including free medication, allowing only payment for diagnosis.

PHF providers also described how important it was to have continuous supplies to provide sustained care to the community:

We give health education which has increased people’s awareness and demand [for service]. Even with malaria being sporadic, the number of patients increased compared to before. People are aware that medication is free. […] Not only for malaria but also for HIV and TB, the medication supply has to be sustainable and continuous for quality service.

##### Remaining gaps

Despite the policy to ensure sustained supplies of medicines, PHFs commonly experienced stockouts of critical medications and supplies, mainly to treat malaria, and of laboratory reagents for TB and malaria testing. Table 4 shows the stockout levels from the HFA.

**Table 4:** Stockouts of selected medicines and products at the PHFs

Products	Reported Stockouts in PHFs (Percent)
**FP commodities** (*n* = 54)	
Oral contraceptive pills	2%
Injectables	6%
Implants	7%
IUCDs	6%
Condoms	15%
**Malaria drugs** (*n* = 50)	
Artemisinin-based combination therapy	44%
Chloroquine	34%
Quinine	54%
Artesunate	58%
Primaquine	50%
**TB drugs** (*n* = 75)	
Rifampin (R) + isoniazid (H) + pyrazinamide (Z) + ethambutol (E)	11%

Widespread opinions that the PHFs sold the free medicines in the market and/or to patients may have led them to get lower priority in receiving the required quantity or consistency of medicines. Some PHFs reported sensing this lack of prioritization from the woreda and regional logistics systems.

Although 57% of the PHFs interviewed expressed satisfaction with the supplies from the woredas, 43% were unsatisfied. Of the 43% unsatisfied, 53% cited irregular supplies, and 47% reported not being a priority for the woreda as the reasons for dissatisfaction. Malaria-sector PHFs expressed the most dissatisfaction. One malaria PHF provider explained:

We submit requests for medicine to government health facilities. A stronger connection with the government HF is needed so that we can get medicines when we request. There are times even when the drugs are available in their stocks at the [public] health facilities but we don’t get them on time.

One RHB representative indicated that even though the PHFs were trained in stock level quantification and were supposed to request medicines and supplies in advance, the PHFs often failed to submit information about the quantities needed early enough:

Private health facilities do not always place the request [for drugs] on time and sometimes ask for supplies at the very end […], we try to arrange from public facilities nearby so the service was not interrupted.

### Health information systems

#### Inclusion of PHFs in the HMIS reporting system

It is required by law that PHFs report to the national HMIS to determine the contributions of PHFs in the provision of services. However, respondents stated that PHFs reporting to the national HMIS is not common practice.

A key impetus for timely reporting that was enforced by the PHSP through an MOU was that without reports, the PHFs would not be able to restock their supply of medicines from government sources. Supported PHFs received training and regular monitoring, which greatly increased their reporting. An RHB informant described the support that they provided to PHFs for the HMIS:

Just like public facilities, proper trainings are provided to PHF representatives on how to use the HMIS system and proper reporting. We identify gaps during supportive supervisions and try to improve their reporting qualities.

##### Remaining gaps

Representatives from PHFs and the RHBs highlighted several challenges in the PHFs’ reporting to the HMIS, including the lack of trained personnel, staff turnover, the reporting burden, and a misunderstanding about the purpose of reporting. The reporting burden varied by area of clinical service. One RHB representative explained:

Similar gaps are [found in] both private and governmental facilities; the HMIS reports are not submitted timely; they do the work and keep registers at the health facilities but do not take reporting seriously. However, this is more common in the PHFs—to submit poor quality data. Data quality gaps include not reporting timely. When you go to the zonal, woreda, and other lower levels, you might find other problems, but these are the problems at the regional level.

Routine reporting for TB and malaria services by the PHFs was relatively straightforward because they are disease-specific and do not require multiple registers or record keeping. Yet even PHFs providing TB services indicated that the availability of reporting forms and changes in the reporting format created issues. One PHF TB representative stated:

Reporting and recording formats are frequently being changed. Sometimes we will be given a single sheet of reporting paper and logbooks, and we were told to duplicate them. This is costing us additional money.

The PHFs noted the most challenges in reporting MNCH and FP services, which require multiple registers and additional workload. In addition, poor data quality, although not unique to PHFs, was stated to be a limitation of reported data. A PHF provider of MNCH services noted:

Regarding the data quality, a deep assessment is required to identify the underlying causes, such as workload, negligence, or lack of knowledge. If I take our facility as an example, the workload is the reason for low data quality not knowledge. It is good to have regular internal monitoring to improve data quality and add more trained staff.

The most significant gap in private sector reporting to the national HMIS was the lack of disaggregation of indicators (except for TB services) by the public health facilities or the sub-regional offices to which the PHFs reported their numbers. The woreda or zonal levels merged the reports from the PHFs with those from the public sector making it impossible to analyze the private sector’s contribution in the HMIS dashboards or reports.

### Human resources

#### Training

The MOH and RHBs developed a workforce within the PHFs that was trained on clinical, laboratory, and logistics, with financial and resource mobilization support by the PHSP. The benefits of the training were shared by a PHF providing malaria services:

Before the training on malaria [detection], we used to send patients with fevers for typhoid screening only, now patients are tested for malaria also as per the guideline.

PHSP respondents reported that clinical and laboratory training, and specialized training, such as basic emergency obstetric and newborn care (BEmONC), which required in-service follow-up mentoring, were resource-intensive and logistically demanding. However, recipients expressed benefitting from the intense training:

There was a gap in up-to-date knowledge on maternal health services between government and private facility staff before we received training on BEmONC. Previously, when staffs from the public facilities talked in different professional meetings, we [private facilities staff] used to get confused as our education was limited from the university training. We now have same knowledge.

Training alone was not enough to deliver the quality services expected from the PHFs. Stakeholders mentioned that supportive supervision and onsite mentorship were efficient ways to improve training quality and motivation for adherence to treatment guidelines.

##### Remaining gaps

The HFA found that 73% of the 106 PHFs perceived that the training they had received was adequate; however, one-quarter of the respondents thought that the training or the refresher training needed further improvement. Despite the inclusion of PHFs in the RHB’s training system, some PHF owners shared that they were not included:

There are government facilities that get training, but private facilities haven’t been involved. Private facilities are providing similar services as the government facilities, yet we are not getting refresher training. A recent example is the family member index training for HIV/AIDS, where we were not invited and didn’t get the training. This is something that has to be corrected.

### Service delivery

The MOH put in place several enabling strategies that served as an impetus to PHFs for service provision, including training and mentoring of providers, improved diagnostic services, access to free or reasonably priced services, and free medications (and free follow-up services). Stakeholders perceived that these strategies contributed to increased demand and service use.

#### Incorporation of PHFs in the national laboratory quality assurance program

One key policy strategy was to link the PHFs with laboratory services for HIV/AIDS, malaria, and TB with the national External Quality Assessment (EQA) program that ensures the quality of laboratory diagnostics. This not only improved the quality of laboratory services but also helped to build trust between the public and private sectors and improved the confidence of the public sector in the quality of services offered by PHFs. An EMLA representative stated:

We have developed a guideline for sample referral linkages across regions and national laboratories among private and public laboratories.

#### Incorporation of PHFs in the laboratory transportation system and referral linkage

In addition to the EQA program, the government included the private sector in the national laboratory sample transportation system to bring samples (for example, HIV viral load, CD4, Gene Xpert testing, and drug-resistant testing for TB) to a network of government-owned regional laboratories. The PHFs were connected to the government postal services to transport specimens to the regional laboratories for advanced laboratory testing. According to stakeholders, this referral linkage benefitted service delivery and demonstrated MOH and RHBs’ commitment to the integration of PHFs in the health system.

##### Remaining gaps

Although the HFA results showed a high coverage of EQA in the past six months, both regional and health facility key informants expressed concerns that the already-stretched regional laboratories might be too overburdened to accommodate the PHFs. Moreover, there were inefficiencies in the transport of laboratory samples and referral linkages with regional laboratories. Stakeholders from the PHFs, PHSP, and Ethiopia Public Health Institute noted that specimen transport to the regional laboratories for further testing did not always work efficiently, and that the government postal system might have limited capacity to cover sample transportation from all PHFs due to time and resource constraints. An RHB representative said:

The postal service does not access all the PHFs. Sometimes the specimen transport from some health facilities was done with public transport. There is a shortage of specimen transportation material, triple packaging materials, etc. at the postal services as well.

### Contributions from PHF to service delivery

PHF stakeholders stated that the PHFs were able to treat additional patients due to the policies that enabled them to have a trained workforce, access to medicines, and overall support from the RHBs and the PHSP. Stakeholders said that the idea of a “one-stop shop” with a diversity of professionals in one place and less waiting time made seeking services from the private sector a preferred option for the community. Some RHB informants perceived that the increased provision of health services by the PHFs decreased the workload at public health facilities. An RHB representative stated:

Our community members when they are sick, they often go to the private health facilities. As they find PHFs as accessible and less time-consuming. By strengthening the private sectors, we are filling the gap in the public sectors, decreasing the load from public facilities, and supporting the health of the community.

#### TB service delivery

The national TB program recognized the PHFs’ contributions in the identification of suspected TB cases and referrals at primary healthcare clinics to reach missed TB cases and it included PHFs in the national strategic plan. A representative from the TB directorate said:

PHFs contribute about 15% of TB case detection in the National TB program, though only 700 (out of 10,000) PHFs are trained and engaged in TB program. Our lesson is to increase the enrolment of PHFs for TB case detection.

Table 5 presents the TB services provided at sampled PHFs supported by the PHSP.

**Table 5:** TB service delivery rendered at PHFs (*n* = 75)

Service	**Number (Percent)**
Trained staff to provide TB treatment	64 (85.3)
Trained staff to conduct acid fast bacilli test	66 (88.0)
Conducts diagnosis	75 (100.00)
Provides treatment	69 (92.0)
Conducts referral	74 (98.7)
Follow-up of referred cases	43 (57.0)
Conducts HIV testing for TB cases	71 (94.7)
Gene Xpert test requested for all TB cases for drug susceptibility testing	43 (57.3)
Contact tracing (responded always or sometimes)	69 (92.0)

#### Malaria service delivery

The MOH malaria prevention and control program was initially resistant to PHFs and delayed approval of the PPM malaria implementation guideline. However, it later expressed appreciation for the PHFs’ engagement. According to the HFA, almost all facilities surveyed (96%) reported screening patients with fever for malaria with blood film; 96% conducted internal quality control either always or sometimes; and 88% reported to the public health emergency management weekly. A high percentage (78%) mentioned experiencing stockouts of at least one malaria drug in the six months before the survey. Table 6 presents the malaria services offered by the PHFs that participated in the HFA.

**Table 6:** Malaria service delivery provided by PHFs (*n* = 50)

Service	**Number (Percent)**
Trained staff to provide malaria treatment	46 (92.0)
Trained staff for malaria microscopy	42 (84.0)
Investigates patients with fever with blood film	48 (96.0)
Reports malaria cases weekly using public health emergency management	44 (88.0)
Conducts internal quality control	48 (96.0)
Regional lab conducts EQA	43 (86.0)
Keep slides for EQA	47 (94.0)
Experienced stockouts of at least one of the anti-malarial drugs in past six months	39 (78.0)

##### Remaining gaps

One of the major challenges according to the key informants was the high staff turnover at the PHFs which affected the availability of trained clinical staff. Trained staff often leave without the proper transfer of clinical knowledge to other staff likely disrupting the provision of services according to national guidelines and protocols across the PHFs. Some PHF owners mentioned that there was also inadequacy in the capacity of clinical staff who provided similar services as a result of the selected number of providers receiving training and mentoring. One of the PHF owners for malaria commented:

We have received training on malaria diagnostics. But, after the lab technician left, there is none […] it would have been better if more, at least two, lab technicians were trained.

Reduced frequency and irregular supportive supervision, mentoring, and supervision on data quality were challenges for quality services. A malaria PHF staff member stated:

Supervision previously helped us to get feedback and solution to our issues and helped improving services. Now, it has stopped and we don’t when we will get supportive supervision.

Another challenge for service delivery was the lack of enthusiasm on the part of private for-profit providers to continue providing certain services, such as TB treatment as they sometimes didn’t perceive any monetary or other incentives. Initial participation in implementing the PPM-TB guidelines by PHFA provided them opportunities for capacity building and capturing a higher number of patients at their facilities. Due to these services, the PHFs also incurred additional costs including uncompensated staff time which might have discouraged PHFs from retaining TB patients for long-term treatment.

### Finance

A variety of financing mechanisms support PPM and PPPH; many rely on external assistance. The PPPH Unit was established through donor support and is currently receiving technical assistance from CHAI. The PPMs were largely dependent on the PHSP bearing the major costs of key activities, such as training, refresher training, trainees’ per diem, and logistics. MOH policies enabled the PHFs to have free medicines and supplies, use government referral facilities for patient referrals, and benefit from the EQA program. The TB PPM is currently supported by the Global Fund to Fight AIDS, Tuberculosis, and Malaria.

It is unclear how the support of PPMs will be financed in the future. In general, the banks in Ethiopia do not have the practice of lending to the health sector because of their lack of knowledge in the financial feasibility of the health sector industry, and the perception that lending to PHFs is riskier than lending to known borrowers with collateral. Using the USAID development credit authority (DCA) mechanism, the PHSP increased access to finance from banks by building the capacity of the private sector to prepare business plans, apply for loans, and manage post-loan monitoring.

The future roles and responsibilities of PPMs in the absence of clear financial support have been raised as a concern. Stakeholders acknowledged that the RHBs have limited human and financial resources to continue the work of managing, mentoring, and supervising the private sector without external support. It was stated that health planning at national and regional levels did not consider the financial support needed for stewardship of the private sector and, therefore, no resources were allocated to the government bodies to support service delivery at the PHFs. One example came from a PHF working in HIV/AIDS. The HIV/AIDS sector’s funding ended early due to the termination of funding from the United States President's Emergency Plan for AIDS Relief (PEPFAR):

When support stopped, the quality of care is affected. We are providing services as per previous training, we are not getting the updated knowledge on HIV/AIDS treatment.

According to PHSP staff and PHF owners, meeting the infrastructure and human resource requirements of stringent health facility standards became difficult for several PHF owners. One of the barriers to achieving the health facility standards was the lack of financing to expand their infrastructure (premises and equipment) and to employ the required number of health professionals. As a result, some owners downgraded their health facility’s level to lower levels of care.

## Discussion

In the past five years, the Ethiopian MOH has opened up to and embarked on a journey to create an enabling environment to foster PPPs [23]. This study used the WHO health systems building blocks to assess the strengths and gaps of PPPHs in Ethiopia supported by the PHSP. It highlighted key milestones for incorporating PHFs in government-led systems, lessons learned, and continuing challenges to expanding implementation.

### Continued gaps in governance and leadership

The WHO has emphasized that accountability of both public and private actors is critical to advancing PPPH; however, accountability structures are context specific [42]. Areas in need of greater accountability identified by this study are: an understanding of how services should be supplied through the MOUs and PPMs; ensuring the availability of adequate resources for PHFs through partnerships with and support from other governmental entities (EFDA, EPSA, EMLA, etc.); performance in the supply of services by monitoring the number of services delivered; receipt of relevant information to monitor performance by strengthening and enforcing HMIS reporting by the PHFs; and enforcement of sanctions through the application of regulatory standards compliance.

Linkages between participants in the PPPH are either tenuous or fragmented, resulting in unclear institutional arrangements. The PPPH Unit, which sits in the Partnership and Collaboration Directorate, has no clear linkages with the different disease-specific directorates that are implementing the PPMs. Although there are legal mandates, frameworks, and a unit at the MOH level, the RHBs work autonomously, without any guiding frameworks or any dedicated units or personnel to oversee the activities of the PHFs. This gap threatens the sustainability of the partnership, especially because the RHBs are overburdened and understaffed.

### The importance of trust

Trust is a central tenet of consensus building and partnership. The importance of trust between public and private actors, the value of consensus building, and the need to find common ground have been emphasized by health system actors engaged in PPPHs across diverse health system contexts [42–44].

Our findings similarly emphasize the importance of mutual trust; however, there was evidence that trust was fragile. The assessment demonstrated that it takes time and evidence to change the negative perceptions of government agencies and staff at all levels about engaging with the private sector. Per its mission, the PHSP focused on continuous dialogue—and perhaps most importantly, through evidence of the private sector’s contributions—for identifying and treating diseases of public health importance. This shifted the government’s perception. The experience of working with the public sector and its systems, and with more transparency and improved regulations, also helped shift perceptions on the private sector side. Yet, mutual trust building between two unequal partners having reservations about each other, especially when government actors hold more power, needs further dedicated work [39]. Any challenges in the support were often viewed with skepticism by both sides.

### Integration of PHFs in government-led systems requires resources

Incorporation of the private sector in government systems—such as the EQA program, laboratory specimen transportation and referral, medicine and commodity supply chains, and the HMIS—formed a platform for sustainable partnership. However, these systems need to function effectively and efficiently, otherwise the PHFs run the risk of interrupting their service delivery. The challenges reported by the private sector in this study have been similarly identified in the public sector concerning coverage of the EQA [45] and issues of stockouts and unreliable supply chains [46, 47]. These functions are already stretched from serving the public sector and incorporating the PHFs has likely created an additional burden.

The PHFs that were part of an MOU have indisputably extended their portfolios and are providing services [48], and to some extent, as the RHB respondents perceived, have reduced the burden on public facilities by sharing the patient load. Training, supportive supervision, and mentoring are essential support systems that are the pillars of quality services for both public and private health facilities in Ethiopia [49–51] and globally. The RHBs should continue offering the PHFs that sign MOUs training, mentoring, and supervision. However, during the period of its support, the PHSP covered much of the costs for training sessions, intensive post training mentorship, and joint supportive supervision. It is critical to identify how the RHBs can continue these activities without external financing.

Recent recommendations about engaging private providers toward UHC have highlighted the costs associated with building public sector capacity and the possibility that governments face tradeoffs in allocating public funds to existing public infrastructure or to the incorporation of private providers in government-led systems [44]. The importance of donor support to PPM collaboration mechanisms for TB PPM has been similarly documented [7]. This study offers similar findings and emphasizes planning for and allocating the human and financial resources required from the public sector to integrate PHFs.

### Service delivery challenges by service area

Although policies and strategies adopted by the MOH have created opportunities for wider partnerships, the assessment found that the implementation of private sector engagement remains limited in scope. There are more than 5,401 private, primary, and lower-level facilities in Ethiopia. They are a huge resource for improving access to essential health services [23]; however, only a handful of PPM implementation guidelines have been developed and only a limited number of PHFs are able to offer treatment for TB, malaria, HIV/AIDS, and limited MCH and FP MNCH services. These facilities serve as a potential resource for improving access to TB, HIV/AIDS, malaria diagnosis and treatment, and MNCH and FP services, and have the potential to be incorporated in the PPM. However, several service delivery challenges need to be overcome. They are summarized below.

In the TB sector, RHBs and the stakeholders expressed concern about the lack of contact tracing and follow-up of referral cases for TB patients, which was low even at government facilities [52]. Although the PHFs mentioned giving patients diagnosed with TB the option to be treated at the PHF or at the nearest government health facility [52], concerns were raised about the number of TB cases being diagnosed increasing in the PHFs, while the proportion of TB cases being treated at the PHFs remained low. That is, TB cases diagnosed at PHFs were often referred to government health facilities for treatment. The lack of aligned incentives is a threat to sustainability, and continuation of the initiation and ongoing treatment of TB at the PHFs. Most of the PHFs targeted by the PHSP were for-profit. They benefited from the Program’s capacity building and ongoing support, but they lacked financial incentives to provide free services. There are examples of incentive-based TB case management through PPP. Ethiopia should explore those options and consider adapting them to the local context [53–55].

For malaria, the government expressed concern about whether the PHFs were strictly following the protocol and current treatment plans [56]. Although PHFs have been providing MNCH and FP services, only a small number of PHFs were enrolled in the PHSP. Although several providers received training in BEmONC and national guidelines, and undoubtedly, a demand for MNCH services increased through the PHFs, the number of cesarean section deliveries was high and seemed to be on the rise [53].

These challenges highlight the need for additional research on the quality of care (QoC) of services provided through PPM and how it compares across service areas. Evidence about QoC in the public versus the private sector is lacking [57]. Previous studies have hypothesized that service quality and responsiveness may be higher at private facilities but that technical quality is possibly lower [15, 58]. A 2016 review found that training and regulation interventions with PHFs may improve QoC; however, the quality of evidence was low and limited [16] and other studies have documented a substantive “know-do” gap that limits the effectiveness of training and standardized guidelines in the private sector [59]. Future research can compare the quality of public and private facilities in this context and can assess the role of the specific interventions and implementation strategies in improving QoC.

### Limitations of the study

This study has several limitations. First, the sampling frame for the HFA was confined to PHFs supported by the PHSP and prioritized PHFs participating toward the end of the program period to ensure avoidance of recall problems by respondents. This limited the collection of experiences from facilities that had graduated from the Program and that had started receiving only government support. Second, the KII and HFA data collection was conducted over the phone due to the COVID-19 pandemic, which limited interactions and participation. As such, the qualitative and quantitative information sometimes lacked in-depth information. Additionally, since about half of the PHFs sent pictures of their current records, it was not feasible to verify some of the self-reported information, such as stockout data. The confirmability of some of the data was not possible and therefore a limitation. However, portions of the records data from the remaining PHFs was triangulated with the pictures sent. Additionally, data collected from some key informant interviews were supported and/or replicated by the data collected from other key informants of the same type (e.g., MOH, PHFAs, etc.). Given this level of credibility, the findings of this study are trustworthy.

Finally, while not a limitation of the study itself, it should be noted that since the study design and data collection tools were developed to meet the specific objectives of assessing PHSP’s performance in leadership and governance, access to medicines, health management information systems, human resources, service provision, and finance, we urge caution in interpreting the findings as an overall assessment of PPPH in general.

## Conclusions

This study explored PPPHs and PPM from the perspectives of stakeholders within the scope of the PHSP in Ethiopia. Analyzed according to WHO health systems building blocks, the findings reveal the achievements and areas of gaps and challenges to sustainability of the partnership. The study identified progress in developing an enabling policy environment, engaging PHFs in service delivery through PPM, integrating PHFs in government systems, and building trust through evidence-informed advocacy. However, challenges remain in improving institutional arrangements for governance at the federal and regional levels, ensuring sustainability of the reforms in the context of an overburdened public sector, continuing to build trust across partners, and ensuring service delivery quality. The lessons offer implementation insights to other low-income country health systems considering how to strengthen stewardship and integration of PHFs in government-led health systems to expand access to essential health services.

Future efforts should emphasize a mechanism that ensures that the private sector is capable, incentivized, and supervised to render continuous high-quality equitable services. Additional research can assess the quality of services provided by the private sector, equity, and economic analysis of PPPH reforms. Continued evidence is needed to assess the specific health system levers that can best use existing private sector capacity toward UHC objectives.

## Data Availability

The dataset supporting the conclusions of this article is publicly available on the University of North Carolina’s Dataverse at the following link: https://dataverse.unc.edu/dataset.xhtml?persistentId=doi:10.15139/S3/ILWJEA.

## Abbreviations

ACIPH: Addis Continental Institute of Public Health
BEmONC: Basic emergency obstetric and newborn care
CHAI: Clinton Health Access Initiative
D4I: Data for Impact
DCA: Development Credit Authority
DOTS: Direct Observed Therapy, short course
EFDA: Ethiopian Food and Drug Administration
EMLA: Ethiopian Medical Laboratory Association
EPSA: Ethiopian Pharmaceutical Supply Agency
EQA: External quality assessment
FMHACA: Food, Medicine and Health Care Administration and Control Authority
FP: Family planning
GOE: Government of Ethiopia
HFA: Health facility assessment
HMIS: Health management information system
IPLS: Integrated pharmaceutical logistics system
JSI: John Snow, Inc.
KII: Key informant interview
LMIC: Low- and middle-income country
MNCH: Maternal, newborn, and child health
MOH: Ministry of Health
MOU: Memorandum of understanding
PHF: Private health facility
PHFA: Private health facility associations
PHSP: Private Health Sector Program
PPM: Public–private mix
PPP: Public–private partnership
PPPH: Public–private partnership in health
QoC: Quality of care
RHB: Regional health bureau
MNCH: Maternal, newborn, and child health
TB: Tuberculosis
UHC: Universal health coverage
USAID: United States Agency for International Development
WHO: World Health Organization
